# DNA-Metabarcoding of Belowground Fungal Communities in Bare-Root Forest Nurseries: Focus on Different Tree Species

**DOI:** 10.3390/microorganisms9010150

**Published:** 2021-01-11

**Authors:** Diana Marčiulynienė, Adas Marčiulynas, Jūratė Lynikienė, Miglė Vaičiukynė, Artūras Gedminas, Audrius Menkis

**Affiliations:** 1 Institute of Forestry, Lithuanian Research Centre for Agriculture and Forestry, Liepų Str. 1, Girionys, LT-53101 Kaunas District, Lithuania; adas.marciulynas@lammc.lt (A.M.); jurate.lynikiene@lammc.lt (J.L.); migle.vaiciukyne@lammc.lt (M.V.); m.apsauga@lammc.lt (A.G.); 2 Department of Forest Mycology and Plant Pathology, Uppsala BioCenter, Swedish University of Agricultural Sciences, P.O. Box 7026, SE-75007 Uppsala, Sweden; audrius.menkis@slu.se

**Keywords:** fungal diversity, community composition, ectomycorrhiza, pathogens, oomycetes, tree seedlings

## Abstract

The production of tree seedlings in forest nurseries and their use in the replanting of clear-cut forest sites is a common practice in the temperate and boreal forests of Europe. Although conifers dominate on replanted sites, in recent years, deciduous tree species have received more attention due to their often-higher resilience to abiotic and biotic stress factors. The aim of the present study was to assess the belowground fungal communities of bare-root cultivated seedlings of *Alnus glutinosa*, *Betula pendula*, *Pinus sylvestris*, *Picea abies* and *Quercus robur* in order to gain a better understanding of the associated fungi and oomycetes, and their potential effects on the seedling performance in forest nurseries and after outplanting. The study sites were at the seven largest bare-root forest nurseries in Lithuania. The sampling included the roots and adjacent soil of 2–3 year old healthy-looking seedlings. Following the isolation of the DNA from the individual root and soil samples, these were amplified using ITS rRNA as a marker, and subjected to high-throughput PacBio sequencing. The results showed the presence of 161,302 high-quality sequences, representing 2003 fungal and oomycete taxa. The most common fungi were *Malassezia restricta* (6.7% of all of the high-quality sequences), *Wilcoxina mikolae* (5.0%), *Pustularia* sp. 3993_4 (4.6%), and *Fusarium oxysporum* (3.5%). The most common oomycetes were *Pythium ultimum* var. *ultimum* (0.6%), *Pythium heterothallicum* (0.3%), *Pythium spiculum* (0.3%), and *Pythium sylvaticum* (0.2%). The coniferous tree species (*P. abies* and *P. sylvestris*) generally showed a higher richness of fungal taxa and a rather distinct fungal community composition compared to the deciduous tree species (*A. glutinosa*, *B. pendula,* and *Q. robur*). The results demonstrated that the seedling roots and the rhizosphere soil in forest nurseries support a high richness of fungal taxa. The seedling roots were primarily inhabited by saprotrophic and mycorrhizal fungi, while fungal pathogens and oomycetes were less abundant, showing that the cultivation practices used in forest nurseries secured both the production of high-quality planting stock and disease control.

## 1. Introduction

In Europe, forest tree planting has increased considerably in recent decades [1,2], thereby increasing the demand for planting stock. The increased planting is primarily due to the commitment in many countries to increase the forest area and/or to rehabilitate degraded forest ecosystems, and thus to reclaim disturbed sites for forestry. This also allows us to maintain and increase biodiversity, and to mitigate the negative effects of global climate change [2,3]. In European forests, tree planting is a principal reforestation practice that also contributes to the sustainability and productivity of forests. Nowadays, most of the planting stock, which is used in forestry, is produced in forest nurseries [4]. The quality of the seedlings produced is one of the critical factors that contributes to successful forest restoration programs [5]. Although the seedling quality may depend on different factors, the associated microbial communities can be of key importance, and may determine the success of the seedlings’ survival, establishment and growth after outplanting [6,7,8].

In nature, healthy and asymptomatic plants cohabit with diverse microbes that form complex microbial consortia and impact plant growth and productivity [9,10]. Several studies have reported a wide range of beneficial effects of microbiota members on plant health, including disease suppression [11,12], the priming of the plants’ immune systems [13], the induction of systemic resistance [14], increased nutrient acquisition [15], increased tolerance to abiotic stresses [16], better adaptation to different environmental conditions [17], and the promotion of root mycorrhization [18]. Among the beneficial microbes, ectomycorrhizal (ECM) fungi are known to provide nutritional benefits to host trees, and may also mitigate negative effects of different abiotic and biotic factors [19]. For example, Zak [20] has postulated several mechanisms by which ECM fungi may provide disease protection to the feeder roots of plants. However, the conditions that often prevail in forest nurseries—namely confined space, high soil fertility, the use of fungicides, and abundant watering—may discourage the ECM colonization of seedling roots [21,22,23]. Such nursery conditions may select for opportunistic ECM fungal species, while limiting root colonisation by ECM fungi present in forest ecosystems [23]. In contrast to ECM fungi, pathogenic fungi or bacteria and plant nematodes may have a negative effect on seedling health, thereby causing abnormal growth or even mortality [24]. Infestations caused by oomycetes, including *Phytophthora* species, can be another threat to seedling production in forest nurseries. Jung et al. [25] demonstrated that nursery seedlings across Europe are commonly infested with a large array of *Phytophthora* spp. Nursery diseases may also have a negative effect on the field performance of outplanted seedlings. Besides this, nursery diseases may also be a threat to local forests when infected seedlings are outplanted, especially in areas where the disease was not present [26]. For example, nursery stock has been shown to be the most common means for the introduction of new *Phytophthora* species into landscapes and habitats worldwide [27,28]. Although many previous studies have linked *Phytophthora* diseases to ornamental plants [25,29,30,31], recent studies suggest that these are also found on native plants produced in forest nurseries [32,33,34].

Interactions between plants and soil microbes are highly dynamic in nature [35,36,37,38], and rhizosphere microbial communities may differ between different plant species [39,40,41], different genotypes within the species [42,43], and between the different developmental stages of a given plant [44,45]. For example, the assessment of fungal communities in the roots of healthy-looking *P. sylvestris* and *P. abies* seedlings in Swedish forest nurseries has showed the dominance of ECM and/or endophytic fungi, but has also revealed some differences in the fungal community composition between the two tree species [46]. Bzdyk et al. [47] demonstrated that the roots of nursery-grown *Fagus sylvatica* and *Q. robur* were inhabited by rather different communities of fungi. By contrast, Beyer-Ericson et al. [48] studied the root-associated fungi of diseased seedlings in Swedish forest nurseries, showing that the commonly-detected fungi were fungal pathogens, including species from the genera *Cylindrocarpon*, *Fusarium, Pythium*, *Botrytis*, *Alternaria* and *Ulocladium*. Similarly, in Norwegian forest nurseries, the roots of diseased seedlings were associated with pathogens from the genera *Pythium* and *Rhizoctonia* [49,50,51], while in Finnish forest nurseries, there were pathogens from the genera *Fusarium*, *Rhizoctonia*, *Pythium* and *Phytophthora* [52,53]. The above studies demonstrate that the root-associated fungal communities in forest nurseries may depend not only on the health status of the tree seedlings, but also on the tree species. Although fungal communities in the healthy and decaying roots of nursery-grown tree seedlings are relatively well understood, studies comparing the belowground fungal communities associated with different tree species are still scarce.

Incidences of locally-occurring, and especially of invasive, forest pathogens have increased exponentially in the last two centuries, causing extensive economic and ecological damage [54]. As nursery seedlings may also serve as a probable source for forest infestation [55,56,57], a better understanding of the fungal and oomycete communities associated with different tree species could be of considerable practical importance. The aim of the present study was to assess the belowground fungal communities of five principle bare-root cultivated tree species in the seven largest forest nurseries in Lithuania using DNA-metabarcoding in order to gain a better understanding of the beneficial and pathogenic fungi and oomycetes, and their potential effects on seedling performance in forest nurseries and after outplanting.

## 2. Materials and Methods

### 2.1. Study Site and Sampling

The study sites were at the seven largest bare-root forest nurseries in Lithuania, namely: Alytus (N 54°24′21.91″, E 24°2′33.27″), Anykščiai (N 55°34′15.07″, E 25°7′3.73″), Dubrava (N 54°50′11.42″, E 24°1′59.23″), Kaišiadorys (54°48′41.97″, 24°33′15.98″), Kretinga (N 56°1′29.82″, E 21°14′2.53″), Panevėžys (N 55°45′32.72″, E 24°30′28.33″) and Trakai (N 54°30′13.05″, E 24°49′46.97″). All of these forest nurseries were situated within a radius of 300 km. Information on each forest nursery, the climate within the area and the soil type is in Table 1. The sampling was conducted between April and May 2018, i.e., at the end of dormancy, but before the time of the seedlings’ outplanting in the forest, by sampling the roots and adjacent soil of 2–3 year old healthy-looking seedlings of *A. glutinosa, B. pendula, P. sylvestris*, *P. abies* and *Q. robur*. The latter seedlings were selected as they represent the principal tree species in the given geographical area. The approximate seedling height was 20 cm for *P. sylvestris* and *P. abies*, 30 cm for *Q. robur,* and 50 cm for *B. pendula* and *A. glutinosa*. During the seedling cultivation in the forest nurseries, fungicides are not used, but fertilizers are applied according to the established routines.

The sampling in each forest nursery included 10 seedling root systems of each tree species, and 10 soil samples that were collected in the vicinity of each collected root system. In total, 350 root samples (7 nurseries × 5 tree species × 10 root samples) and 350 soil samples (7 nurseries × 5 tree species × 10 soil samples) were collected. For the collection of the roots, seedlings were randomly selected, their roots were excavated, they were gently shaken to remove the larger particles of soil, and they were cut off from the shoots. The soil samples (ca. 100 g) were taken using a 2 cm diameter soil core down to a 20 cm depth. The soil core was carefully cleaned between taking different samples. The collected root and soil samples were individually placed into plastic bags, labelled, transported to the laboratory and stored at −20 °C before being further processed.

In the laboratory, the roots were carefully washed in tap water in order to remove any of the remaining soil, and the fine roots with root tips were separated from the coarse roots (which were discarded). The fine roots were cut into ca. 1 cm-long segments, and each forest nursery and tree species was pooled together, resulting in a total of 35 root samples (7 nurseries × 5 tree species). The soil samples were sieved (mesh size 2 × 2 mm) in order to remove larger particles and roots, and each forest nursery and tree species was pooled together, resulting in a total of 35 samples (7 nurseries × 5 tree species).

### 2.2. DNA Isolation, Amplification and Sequencing

The principles of the DNA work followed the study by Marčiulynas et al. [59]. No surface sterilization of the roots was carried out. Prior to the isolation of the DNA, each sample (soil or roots) was freeze-dried (Labconco FreeZone Benchtop Freeze Dryer, Cole-Parmer, Vernon Hills, IL, USA) at −60 °C for two days. After the freeze-drying, ca. 0.03 g dry weight of each root sample was placed into a 2-mL screw-cap centrifugation tube together with glass beads. All of the samples were homogenized using a Fast prep shaker (Montigny-le-Bretonneux, France). The DNA from the roots was isolated using CTAB extraction buffer (0.5 M EDTA pH 8.0, 1 M Tris-HCL pH 8.0, 5 M NaCl, 3% CTAB) followed by incubation at 65 °C for 1 h. After the centrifugation, the supernatant was transferred to a new 1.5-mL Eppendorf tube, mixed with an equal volume of chloroform, centrifuged at 13,000 rpm for 8 min, and the upper phase was transferred to new 1.5-mL Eppendorf tubes. Then, an equal volume of 2-propanol was used to precipitate the DNA into a pellet by centrifugation at 13,000 rpm for 20 min. The pellet was washed in 500 μL 70% ethanol, dried, and dissolved in 50 μL sterile milli-Q water. Unlike the roots, ca. 1 g of freeze-dried soil was used for the isolation of the DNA from each sample using a NucleoSpin^®^Soil kit (Macherey-Nagel GmbH & Co. Duren, Germany) according to the manufacturer’s recommendations. Following the isolation of the DNA, the DNA concentration in the individual samples was determined using a NanoDrop™ One spectrophotometer (Thermo Scientific, Rodchester, NY, USA). The amplification of the ITS rRNA region was achieved using a forward primer ITS6 [60] and barcoded universal primer ITS4 [61]. This primer pair was shown to amplify both fungi and oomycetes [60]. The polymerase chain reaction (PCR) was performed in 50 μL reactions, and consisted of the following final concentrations: 0.02 ng/μL template DNA, 200 μM dNTPs, 750 μM MgCl_2_, 0.025 μM DreamTaq Green polymerase (5 U/μL) (Thermo Scientific, Waltham, MA, USA), and 200 nM of each primer; sterile milli-Q water was added in order to make up the final volume (50.0 μL) of the reaction. The amplifications were performed using a Applied Biosystems 2720 thermal cycler (Applied Biosystems, Foster City, CA, USA). The PCR program started with an initial denaturation step at 95 °C for 2 min, followed by 35 cycles of 95 °C for 30 s, and annealing at 55 °C for 30 s and 72 °C for 1 min, followed by a final extension step at 72 °C for 7 min. The PCR products were assessed using gel electrophoresis on 1% agarose gels stained with GelRed (Biotium, Fremont, CA, USA). The PCR products were purified using 3 M sodium acetate (pH 5.2) (Applichem GmbH, Darmstadt, Germany) and 96% ethanol mixture (1:25). After the quantification of all of the PCR products using a Qubit fluorometer 4.0 (Life Technologies, Stockholm, Sweden), they were pooled in an equimolar mix and sequenced using a PacBio platform and one Sequel SMRT cell at a SciLifeLab facility in Uppsala, Sweden.

### 2.3. Bioinformatics

The sequences were filtered and clustered using the Sequence Clustering and Analysis of Tagged Amplicons (SCATA) next-generation sequencing (NGS) pipeline (http://scata.mykopat.slu.se). The sequences were filtered for quality, removing short sequences (<200 bp), sequences with low mean read quality (Q < 20), primer dimers, and homopolymers, which were collapsed to three base pairs (bp) before clustering. The sequences were screened for primers and sample-identifying barcodes, and those sequences that were missing a barcode or primer were removed. The sequences were clustered into different taxa by single linkage clustering, with a 2.0% maximum distance allowed for the sequences to enter the clusters. The fungal taxa were taxonomically classified using a Ribosomal Database Project (RDP) pipeline classifier (https://pyro.cme.msu.edu/index.jsp), and sequences showing <80% similarity to the phylum level were considered to be of non-fungal or oomycete origin, and were excluded from further analyses. All of the fungal and oomycete sequences were taxonomically identified using the GenBank (NCBI) database and the Blastn algorithm (Table S1). The following criteria were used for the identification: sequence coverage > 80%, similarity to taxon level 98–100%, and similarity to genus level 94–97%. Sequences not matching these criteria were considered unidentified, and were given unique names. Representative sequences of the fungal and oomycete non-singletons are available from GenBank under accession numbers MW214720–MW216333.

### 2.4. Statistical Analysis

The rarefaction analysis was performed using Analytical Rarefaction v.1.3, which is available at http://www.uga.edu/strata/software/index.html. The differences in the richness of the fungal and oomycete operational taxonomic units (OTUs) in the roots and soil among the different tree species were compared using the nonparametric chi-square test [62]. As each of the datasets was subjected to multiple comparisons, the confidence limits for the *p*-values of the chi-square tests were reduced the corresponding number of times required by the Bonferroni correction [63]. The Shannon diversity index, the qualitative Sørensen similarity index, and non-metric multidimensional scaling (NMDS) in Canoco v.5.02 (Microcomputer Power, Ithaca, NY, USA) [62,64] were used in order to characterise the diversity and composition of the fungal communities. A MANOVA in Minitab v. 18.1 (Pennsylvania State University, State College, Pennsylvania, PA, USA) was used in order to evaluate the degree of separation (along NMDS axes 1 and 2) between the fungal communities in the different types of samples (roots and soil) and among the different tree species. For each type of sample (roots or soil), the nonparametric Mann-Whitney test in Minitab was used to test whether the Shannon diversity index differed among the different tree species. The assignment of ecological roles was based on FUNGuild [65].

## 3. Results

A total of 305,139 sequences was generated by the PacBio sequencing. The quality filtering showed the presence of 161,302 (52.9%) high-quality sequences, while the remaining 143,837 (47.1%) low quality sequences were excluded from further analyses. The clustering of the high-quality sequences showed the presence of 3564 non-singleton contigs representing different OTUs. Singletons were excluded. The taxonomic classification showed that 2003 (56.2%) of the OTUs represented fungi (Table S1 and Table 2), while 1561 (43.8%) non-fungal OTUs were excluded. Among all of the fungal OTUs, 390 (19.5%) were identified to the species level, 345 (17.2%) were identified to the genus level, and 1268 (63.3%) were identified only to a higher taxonomic level.

**Figure 1:**
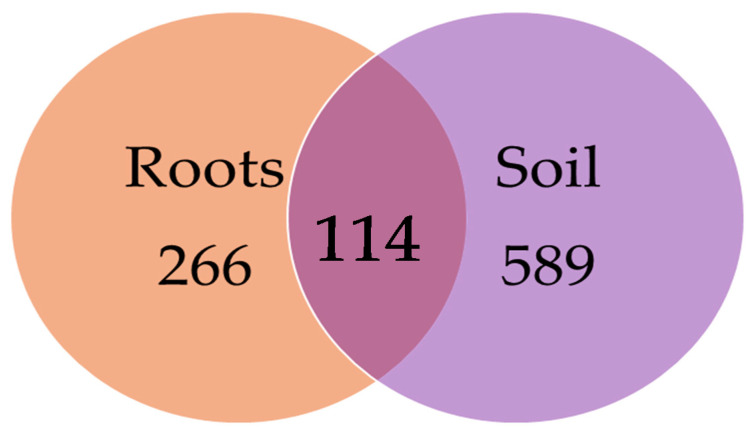
Venn diagram showing the diversity of the fungal OTUs found in the seedling roots and soil, and the number of fungal OTUs shared between both types of samples. The samples from different tree species and forest nurseries are combined.

Of the 2003 fungal OTUs (all of which identified at least to the phylum level, Table S1) across all of the soil and root samples, Ascomycota was the most dominant phylum, which accounted for 50.4% of all of the fungal OTUs, followed by Basidiomycota (31.4%), Mucoromycota (6.2%), Chytridiomycota (6.0%), Oomycota (3.4%) and Zoopagomycota (2.0%). Entorrhizomycota, Blastocladiomycota, Cryptomycota, Zygomycota, Olpidiomycota and Blastoclamidiomycota were also detected, but in a very low proportions (each < 0.2%). Among all of the fungal OTUs (different tree species and nurseries combined), 266 (13.3%) were exclusively found in roots, 589 (29.4%) were exclusively found in the soil, and 1148 (57.3%) were shared between both types of samples (Figure 1).

Information on the 30 most common fungal OTUs—representing 62.03% of all of the high-quality fungal sequences in the roots and 38.95% of all of the high-quality fungal sequences in the soil of the different tree species—is in Table 3. The most common fungi (all samples combined) were *Malassezia restricta* (6.7% of all of the high-quality sequences), *Wilcoxina mikolae* (5.0%), *Pustularia* sp. 3993_4 (4.6%), *Fusarium oxysporum* (3.5%), *Tomentella* sp. 3993_7 (2.6%) and *Suillus luteus* (1.3%) (Table 3). Among the 30 most common fungal OTUs, five OTUs represented plant pathogens, including *F. oxysporum*, *Dactylonectria macrodidyma* (1.1%), *Fusarium solani* (0.9%), *Pestalotiopsis* sp. 3993_40 (0.8%), and *Fusarium* sp. 3993_41 (0.8%) (Table 3). The phylum Oomycota was represented by 68 OTUs, which comprised 1.5% of all of the high-quality sequences in the roots, and 3.2% of all of the high-quality sequences in the soil. Among these, there were 35 (51.5%) OTUs of *Pythium* spp., 4 (5.9%) of *Phytophthora* spp., 3 (4.4%) of *Phytopythium* spp., 2 (2.9%) of *Hyaloperonospora* spp., 2 (2.9%) of *Peronospora* spp., and 1 (1.5%) of *Pythiogeton* sp., while 21 (30.9%) could not be identified to the species or genus level (Table S1). Information on the 30 most common oomycete OTUs is in Table 4. The most common oomycetes were *Pythium ultimum* var. *ultimum* (0.6% of all of the high-quality sequences), *Pythium heterothallicum* (0.3%), *Pythium spiculum* (0.3%), *Pythium sylvaticum* (0.2%), *Pythium irregulare* (0.2%) and *Peronospora* sp. 3993_148 (0.1%) (Table 4).

**Figure 2:**
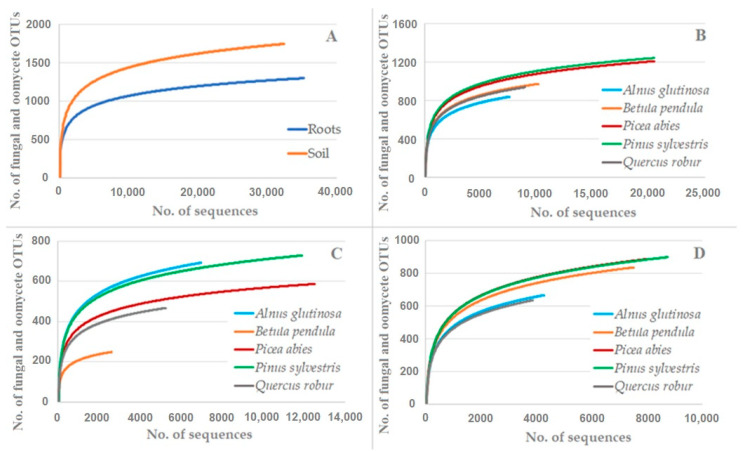
Rarefaction curves showing the relationship between the cumulative number of fungal and oomycete OTUs and the number of ITS rRNA sequences compared: (**A**) roots vs. soil; (**B**) the different tree species (roots and soil samples combined); (**C**) root samples of the different tree species; and (**D**) soil samples of the different tree species.

The rarefaction analysis showed that the number of fungal and oomycete OTUs did not reach species saturation (Figure 2). When the same number of fungal and oomycete sequences had been taken, the richness of the fungal and oomycete OTUs was significantly lower in the roots than in the soil (tree species and nurseries combined) (*p* < 0.0001) (Figure 2A). A similar comparison among the different tree species (root and soil data combined) showed that the deciduous tree species (*A. glutinosa*, *B. pendula* and *Q. robur*) had a significantly lower richness of fungal and oomycete OTUs taxa compared to the coniferous tree species (*P. abies* and *P. sylvestris*) (*p* < 0.0001) (Figure 2B). A higher variation in the richness of the fungal and oomycete OTUs was observed when the root and soil samples were analysed separately (nurseries combined) (Figure 2C,D). For example, in the roots, the richness of the fungal and oomycete OTUs was significantly lower compared *B. pendula* vs. other tree species (*p <* 0.0001), *Q. robur* vs. *A. glutinosa*, *P. abies* or *P. sylvestris* (*p* < 0.0001), and *P. abies* vs. *A. glutinosa* or *P. sylvestris* (*p* < 0.02) (Figure 2C). In a similar comparison, *P. sylvestris* and *A. glutinosa* did not differ significantly from each other (*p* > 0.05) (Figure 2C). In the soil, the richness of the fungal and oomycete OTUs was significantly lower compared to *A. glutinosa* vs. other tree species (*p* < 0.0001) (except *A. glutinosa* vs. *Q. robur*, *p* > 0.05) and *Q. robur* vs. *B. pendula*, *P. abies* or *P. sylvestris* (*p* < 0.0001), while *B. pendula*, *P. abies* and *P. sylvestris* did not differ significantly from each other (*p* > 0.05) (Figure 2D).

**Figure 3:**
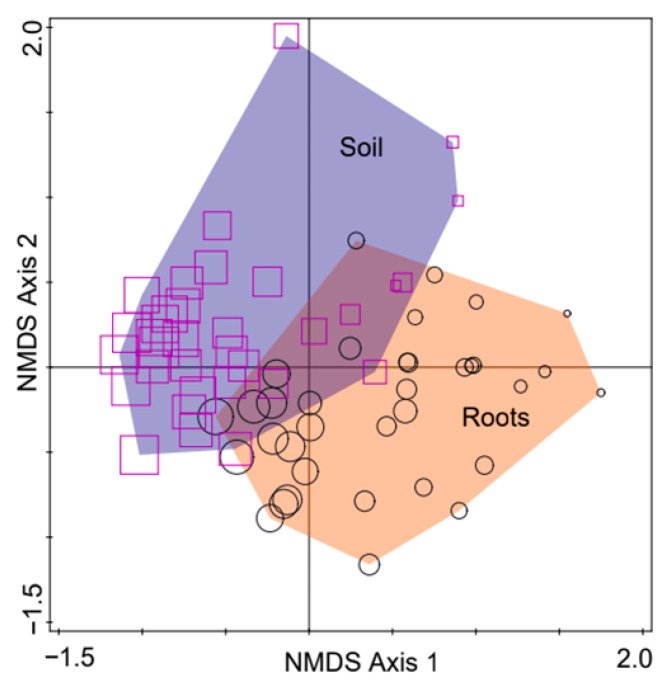
Nonmetric multidimensional scaling (NMDS) of the fungal and oomycete communities in the roots and soil of the five tree species grown in the forest nurseries. Each point (circles for the roots and squares for the soil) represents a separate sample of different tree species, and the size of each point reflects the relative richness of the fungal and oomycete OTUs. The NMDS of the fungal and oomycete communities explained 52.8% of the variation on Axis 1 and 26.8% of the variation on Axis 2.

**Figure 4:**
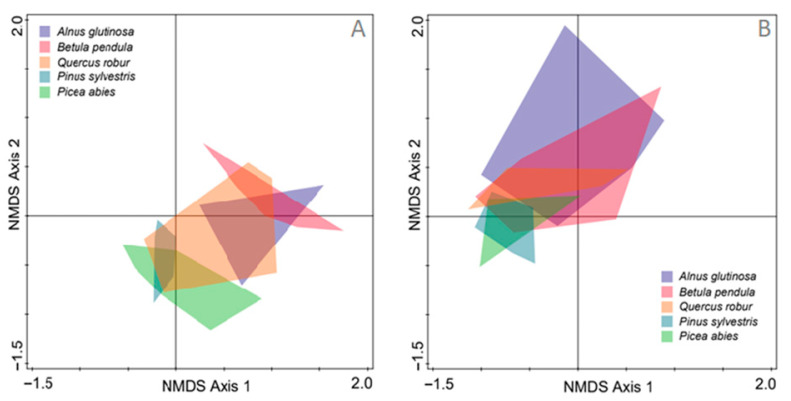
NMDS of the fungal and oomycete communities in the roots (**A**) and soil (**B**) of the five tree species grown in the forest nurseries. The data from the different forest nurseries are combined.

The non-metric multidimensional scaling of the fungal and oomycete communities showed a partial separation of the root and soil samples (tree species and nurseries combined) (Figure 3). The MANOVA showed that this separation was statistically significant (*p* < 0.0001) (Figure 3). For both the root and soil samples, a higher degree of separation of the fungal communities was between the coniferous tree species (*P. abies* and *P. sylvestris*) and the deciduous tree species (*A. glutinosa*, *B. pendula* and *Q. robur*) (Figure 4A,B). The MANOVA showed that the fungal and oomycete communities in the roots of *P. abies* and *P. sylvestris* differed significantly from those in *A. glutinosa* and *B. pendula* (*p <* 0.0001), while the fungal community in the roots of *Q. robur* were similar to all of the other tree species (Figure 4A). A similar comparison of fungal communities between *P. abies* and *P. sylvestris,* and between *A. glutinosa* and *B. pendula* showed that these did not differ significantly from each other (*p* > 0.05), respectively. In the soil, the fungal communities did not differ significantly among the different tree species (*p* > 0.05) (Figure 4B).

**Figure 5:**
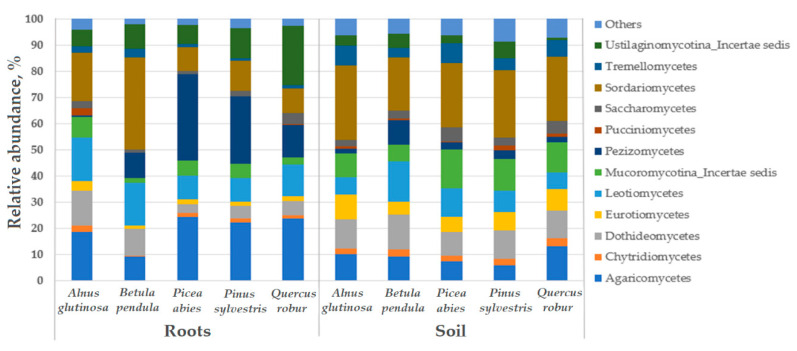
Relative abundance of the fungal classes in the roots and soil of the different tree species, and the other fungal classes that presented with a relative abundance of <1%. The data from the different forest nurseries are combined.

A total of 31 fungal classes was detected. A comparison among the different tree species showed that the relative abundance of fungal classes was more uniform among the soil samples than among the root samples (Figure 5). Nevertheless, in both the root and soil samples, *Sordariomycetes*, *Leotiomycetes*, *Dothideomycetes* and *Agaricomycetes* were the most common (Figure 5). An exception was the class *Pezizomycetes*, which showed a high relative abundance in the roots of *P. abies* and *P. sylvestris*, while *Ustilaginomycotina_Incertae sedis* showed a high relative abundance in the roots of *Q. robur* (Figure 5).

**Figure 6:**
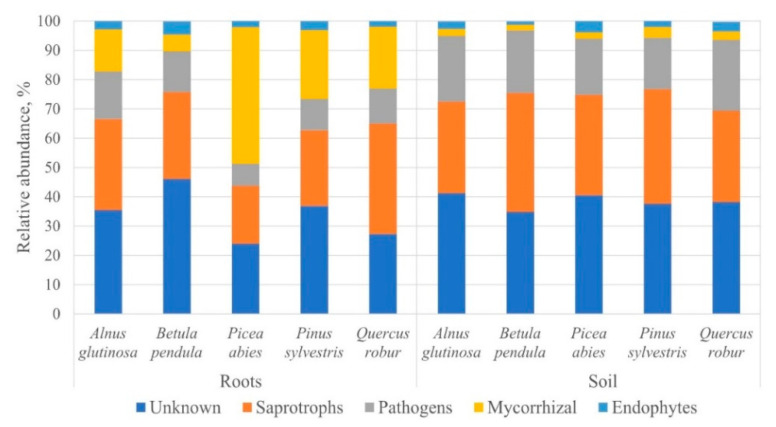
Ecological roles (shown as a proportion of the high-quality sequences) of the fungal and oomycete OTUs detected in the roots and soil of the different tree species. The data from the different forest nurseries are combined.

In the different tree species, the Sørensen similarity index of the fungal and oomycete communities was moderate, and ranged between 0.35 and 0.55 among the root samples, and between 0.43 and 0.56 among the soil samples. The Shannon diversity (H) index of the fungal and oomycete communities was high in both the root and soil samples (Table 2). The Mann-Whitney test showed that the Shannon diversity index did not differ significantly among the root or soil samples of the different tree species (*p* > 0.05). The assignment of fungal and oomycete ecological roles (nurseries combined) revealed a higher variation in the relative abundance among the root samples than among the soil samples of the different tree species (Figure 6). In the roots, the most common fungal and oomycete OTUs were of unknown ecological roles (23.9–46.1%, a range represents different tree species), followed by saprotrophs (19.6–37.7%), mycorrhizal fungi (5.7–46.6%), and pathogens (7.5–16.3%), and the least common were endophytes (1.9–4.5%) (Figure 6). Similarly, in the soil, the most common fungal and oomycete OTUs were unknown (34.7–41.2%), followed by saprotrophs (31.2–40.7%), pathogens (17.6–24.2%), and endophytes (1.8–3.6%), and the least common were mycorrhizal fungi (1.2–3.8%) (Figure 6).

## 4. Discussion

In the present study, a comparison of the five economically-important tree species cultivated under similar conditions in bare-root forest nurseries provided valuable insights into the specificity of the associated fungal and oomycete OTUs. Firstly, the results demonstrated that the seedling roots and the rhizosphere soil were inhabited by a high diversity of fungal and oomycete OTUs (Figure 1 and Figure 2, Table 2), thereby corroborating previous observations that the belowground habitat in forest nurseries supports species-rich communities of fungi [66]. Interestingly, the detected richness of the fungal OTUs can be comparable to those present in the forest stands of the same geographical area [59], even though the rarefaction analysis showed that the observed richness of fungal OTUs can potentially be higher with increased sequencing effort (Figure 2). Secondly, our results revealed that the diversity and composition of the fungal and oomycete communities were partly dependant on the substrate (roots or soil) and/or on the host tree species (Figure 1, Figure 2, Figure 3, Figure 4 and Figure 5). As a result, there was generally a higher richness of fungal and oomycete OTUs in the soil than in the seedling roots, which was probably due to a higher heterogeneity of the soil environment compared to the roots [67], even though the intensive soil preparations (e.g. plowing and harrowing) in bare-root forest nurseries may have a homogenising effect on the soil’s fungal communities (Figure 4, Figure 5 and Figure 6). The segregation of the fungal and oomycete communities between the root and soil samples (Figure 3) was likely influenced by the host tree species, owing to a higher degree of modification of the associated fungal and oomycete communities in the roots than in the soil. In agreement with this, several studies have shown that plants may modify the structure of the associated microbial communities in their roots [68,69]. By contrast, the fungal communities in the soil can only be indirectly controlled by plants through the release of organic compounds that may contribute to the unique rhizosphere nutrient pool which is accessible to the soil microorganisms [70,71,72].

The coniferous tree species (*P. abies* and *P. sylvestris*) generally showed a higher richness of fungal and oomycete OTUs, and a rather distinct community composition compared to the deciduous tree species (*A. glutinosa*, *B. pendula* and partially *Q. robur*) (Figure 2 and Figure 4). These distinctions were more apparent among the fungal and oomycete communities in the roots, but were expressed less in the soil (Figure 4, Figure 5 and Figure 6). As certain root-associated fungi can be host-dependent, and some can even be host-specific [73,74,75,76,77], this demonstrates the relative importance of the host [78]. For example, Ishida et al. [79] showed that taxonomically-close host species harbour similar communities of mycorrhizal fungi. In agreement with this, it appears that mycorrhizal fungi play a key role in shaping the fungal communities in the roots of different tree species, as the abundance of fungi of other ecological roles was rather similar among the different tree species and substrates (roots or soil) (Figure 6). The latter may suggest that fungi of unknown ecological roles, saprotrophs, pathogens and endophytes generally possess a lower host or substrate specificity than mycorrhizal fungi; thus, the former were likely often represented by fungal generalists. Furthermore, in agreement with the previous studies on fungal communities in forest nurseries [7,46,80], the results have shown the dominance of fungal OTUs belonging to Ascomycota (50.4%) and Basidiomycota (31.4%). A higher relative abundance of ascomycetes in the soil and roots from all of the forest nurseries may reflect their better adaptation to a highly-transformed forest nursery environment compared to basidiomycetes. Indeed, members of Ascomycota have been shown to dominate on sites following site disturbances [81].

Among the dominant fungi, *Malassezia restricta* was shown to be exceedingly widespread and ecologically diverse in the environmental samples [82]. It was found in deep-sea sediments [83], hydrothermal vents [84], stony corals [85], Antarctic soils [86,87], on the exoskeleton of soil nematodes [88], and on various plant roots [89]. A recent study has also indicated that *M. restricta* is one of the most frequently-occurring species in the irrigation water of forest nurseries [59]. Despite many investigations, remarkably little is known about the impact of *M. restricta* on plant health. *Wilcoxina mikolae,* which was the second most commonly-detected fungus, was found in different forest nurseries, tree species and substrates (roots and soil) (Table 3), thereby showing a broad ecological niche. In agreement with this, this mycorrhizal symbiont was commonly reported in association with the roots of forest nursery seedlings [90,91]. Besides this, it was shown that *Wilcoxina* fungi can reduce the negative effect of salt stress on the plants [92] and support tree growth in high-altitude marginal habitats [93]. Its common occurrence in forest nurseries and on different hosts raises the question of its potential effect on seedling performance in forest nurseries, but such information is scarce. For example, Smaill and Walbert [94] showed that, on the roots of *Pinus radiata* seedlings, the abundance of *Wilcoxina* increases with increased applications of fertilizers and fungicides. Jones et al. [95] found that seedlings colonised by *Wilcoxina* showed an increased accumulation of ^15^N. *Suillus luteus* was another mycorrhizal fungus commonly detected in forest nurseries (Table 3). *Suillus* spp. are known as pioneer fungi, occurring in association with *Pinus* spp. in forest nurseries and in newly established forest plantations [6,96]. *Pinus sylvestris* seedlings inoculated with *S. luteus* were shown to have significantly better survival and growth rates after outplanting compared to controls [97], thereby demonstrating that this fungus can benefit the host trees.

Although the fungal communities were dominated by saprotrophs (Figure 6, Table S1), pathogens were also detected, indicating their potential threat to the plants. *Fusarium oxysporum* was the most commonly detected pathogen (Table 3), and it is known as one of the most destructive soil-borne pathogens, causing seedling diseases in forest nurseries worldwide [98]. *Fusarium solani* was also detected, but at lower proportions (Table 3). It is often found on dead organic matter, but under certain conditions it can cause disease in various hosts [99]. *Dactylonectria macrodidyma* (previously *Neonectria macrodidyma*) was also commonly recorded both in the root and soil samples of different tree seedlings (Table 3). *Dactylonectria macrodidyma* was shown to be an economically-important pathogen in forest nurseries [48,52,100,101,102]. Interestingly, the above-mentioned fungal pathogens showed generally low host or habitat specificity, but their relative abundance was often higher in the soil than in the roots of the different tree species (Table 3).

Oomycetes represent one of the most problematic groups of disease-causing microorganisms in different growing environments, including forest nurseries [25]. They can also cause diseases in different hosts, including trees, ornamental plants, and crops [103]. In the present study, oomycetes were often more abundant in the soil than in the seedlings’ roots (Table 4), suggesting that, in healthy roots, their development was largely restricted. The oomycetes that were most common in this study (Table 4) are also known to cause seedling diseases in forest nurseries, including taxa such as *P. ultimum* var. *ultimum*, *P. heterothallicum* [104,105] and *P. spiculum* [106,107,108]. *Pythium spiculum* was previously detected in the feeder roots and in the rhizosphere soil of declining oaks [109], but information on its pathogenicity to oaks is limited [106]. Among other oomycetes, *P. irregulare* is known to be associated with more than 200 host plants, and can cause root rot in deciduous and coniferous trees [110]. Interestingly, *Peronospora* sp. 3993_148 was exclusively detected in oak roots (Table 4), which may be due to the fact that representatives of this genus are known to cause downy mildew disease [111]. Among the 30 most common oomycetes, only two *Phytophthora* species were detected, i.e., *Phytophthora pseudosyringae* and *Phytophthora fragariae* (Table 4). *Phytophthora pseudosyringae* is known to cause root and collar rot in deciduous trees [112]. *Phytophthora fragariae* is a root pathogen that causes red stele disease in strawberries [113,114], but in the present study, it was associated with the soil and roots of *A. glutinosa,* and with the roots of *B. pendula* (Table 4).

## 5. Conclusions

The results demonstrated that the seedling roots and the rhizosphere soil in bare-root forest nurseries support a high richness of fungal taxa, which can be comparable to those present in forest stands.

Although the fungal communities in the roots were generally different between coniferous and deciduous tree species, the corresponding fungal communities in the soil were similar, thereby showing the relative importance of fungal generalists. The seedling roots were primarily inhabited by saprotrophic and mycorrhizal fungi, while fungal pathogens and oomycetes were less abundant, showing that the cultivation practices used in forest nurseries secured both the production of high-quality planting stock and disease control.

## Figures and Tables

**Table 1:** Characteristics of the seven largest bare-root forest nurseries in Lithuania that were sampled in the present study.

Forest Nursery	Area of the Nursery, ha	Total No. of Seedlings Produced, Millions	No. of Seedlings Produced by Tree Species, Millions					Mean Monthly Temperature, °C *	Mean Monthly Precipitation, mm *	Soil Type
			*Alnus glutinosa*	*Betula pendula*	*Picea abies*	*Pinus sylvestris*	*Quercus robur*			
**Alytus**	23.1	4.7	0.2	0.4	2.6	1.1	0.3	10.5	49.6	Nc
**Anykščiai**	34.0	5.6	0.3	0.3	4.6	0.2	0.2	10.1	38.2	Nc
**Dubrava**	56.3	7.1	0.3	0.2	5.2	1.2	0.2	10.2	64.8	Nc
**Kaišiado-rys**	27.0	4.6	0.2	0.2	3.6	0.2	0.3	9.9	52.1	Nc
**Kretinga**	66.0	5.5	0.1	0.1	4.4	0.4	0.2	8.8	61.6	Nc
**Panevėžys**	66.0	8.8	0.4	0.3	7.4	0.3	0.5	9.8	46.0	Lc
**Trakai**	31.4	5.1	0.1	0.2	3.2	1.3	0.1	10.3	41.0	Nb

* The climate data is for the period of sampling, i.e., April–May 2018. the soil type: Nb—oligotrophic mineral soils of normal humidity; Nc—mesotrophic mineral soils of normal humidity; Lc—eutrophic gleyic sandy loam [58].

**Table 2:** Generated high-quality fungal sequences and the detected diversity of the fungal taxa in the roots and soil of the five different tree species from the seven forest nurseries in Lithuania. The data from the different forest nurseries are combined.

Samples	Tree Species	No. of Fungal Sequences/Taxa	Shannon Diversity Index H ^a^
**Roots**	*Alnus glutinosa*	6979/691	2.16–4.33
	*Betula pendula*	2620/256	1.93–4.09
	*Picea abies*	12,532/591	1.75–4.28
	*Pinus sylvestris*	11,910/718	2.62–4.32
	*Quercus robur*	5270/453	2.14–4.23
**Total Roots**		39,311/1414	
**Soil**	*Alnus glutinosa*	4292/664	3.28–5.03
	*Betula pendula*	7525/836	2.57–5.20
	*Picea abies*	7961/873	3.28–4.89
	*Pinus sylvestris*	8745/881	3.56–4.69
	*Quercus robur*	3862/646	3.83–5.17
**Total Soil**		32,385/1737	
**All**		71,696/2003	

^a^ The Shannon diversity index H column shows a variation among the different forest nurseries.

**Table 3:** Occurrence and relative abundance of the 30 most common fungal OTUs (show as a proportion of all of the high-quality fungal sequences) in the roots and soil of the five tree species that were bare-root cultivated in the forest nurseries. The data from the different forest nurseries are combined.

OTU	Phylum	Reference	Similarity,% ^a^	*Alnus glutinosa*		*Betula pendula*		*Picea abies*		*Pinus sylvestris*		*Quercus robur*		All	All	Total%
				Roots%	Soil%	Roots%	Soil%	Roots%	Soil%	Roots%	Soil%	Roots%	Soil%	Roots%	Soil%	
*Malassezia restricta*	Basidiomycota	CP030254	727/728 (99)	5.79	3.59	8.02	4.96	6.57	2.85	10.43	6.00	20.75	0.85	9.01	4.05	6.70
*Wilcoxina mikolae*	Ascomycota	KU061020	592/593 (99)	0.11	0.09	0.38	0.07	22.28	0.95	3.82	1.46	0.88	0.08	8.82	0.67	5.03
*Pustularia* sp. 3993_4	Ascomycota	MF352743	513/518 (99)	-	0.26	0.15	4.52	7.50	0.04	15.53	0.03	0.82	-	7.56	1.10	4.56
*Fusarium oxysporum*	Ascomycota	GU136492	547/547 (100)	2.41	5.24	0.73	4.44	3.28	4.81	2.99	4.63	1.91	2.54	2.72	4.46	3.53
*Tomentella* sp. 3993_7	Basidiomycota	KX095160	648/663 (98)	0.01	0.05	-	0.07	14.20	0.15	0.07	0.01	0.15	0.00	4.80	0.06	2.60
*Suillus luteus*	Basidiomycota	KU721223	688/688 (100)	-	0.02	0.04	0.00	0.06	0.01	6.90	0.77	0.12	0.08	2.23	0.22	1.30
*Mycoarthris corallina*	Ascomycota	AH009124	467/473 (99)	6.53	0.44	0.57	9.13	0.02	0.82	0.06	0.11	0.09	0.16	1.30	2.43	1.82
*Paraphaeosphaeria sporulosa*	Ascomycota	KY977581	594/594 (100)	1.83	1.49	0.53	1.62	1.29	2.42	1.22	3.90	0.52	1.11	1.25	2.36	1.76
*Chaetomium cochliodes*	Ascomycota	KT895345	570/570 (100)	0.42	2.10	0.27	1.73	1.51	3.13	1.20	3.56	0.40	1.32	1.02	2.57	1.74
*Pleotrichocladium opacum*	Ascomycota	NR155696	545/549 (99)	0.44	0.40	28.66	0.54	0.80	0.78	0.42	0.29	0.79	0.54	2.57	0.51	1.61
*Halenospora varia*	Ascomycota	AJ608987	538/538 (100)	1.09	0.91	4.77	0.43	1.39	1.88	1.39	1.44	3.71	1.58	1.78	1.26	1.54
*Tomentella sublilacina*	Basidiomycota	HM189981	648/662 (98)	12.94	1.28	0.04	0.00	0.06	-	0.03	0.01	0.18	0.05	2.47	0.18	1.41
*Umbelopsis vinacea*	Mucoromycota	KC489498	628/633 (99)	0.46	0.91	0.42	0.33	1.39	3.49	1.11	2.53	0.27	0.13	0.96	1.75	1.33
*Cladosporium cladosporioides*	Ascomycota	MG664765	547/547 (100)	4.28	0.70	1.22	4.16	0.22	0.14	0.49	0.27	1.12	0.54	1.22	1.23	1.22
*Suillus granulatus*	Basidiomycota	AJ272409	677/678 (99)	-	-	-	-	0.01	-	6.83	0.01	0.03	-	2.18	0.00	1.17
*Solicoccozyma terricola*	Basidiomycota	KY558367	641/641 (100)	0.50	2.89	0.15	1.10	0.26	3.52	0.29	1.53	0.12	1.84	0.29	2.14	1.15
*Phialocephala fortinii*	Ascomycota	AY078131	560/561 (99)	0.96	0.19	0.27	0.09	1.61	1.42	2.34	0.35	1.22	0.05	1.59	0.50	1.08
*Tuber* sp. 3993_24	Ascomycota	KT215193	646/646 (100)	0.03	0.02	0.04	0.00	2.35	0.06	2.64	0.00	5.20	0.03	2.10	0.02	1.13
*Dactylonectria macrodidyma*	Ascomycota	JN859422	541/541 (100)	0.63	2.10	0.38	0.69	0.46	0.90	2.17	0.89	1.22	1.04	1.10	1.03	1.06
*Mortierella* sp. 3993_26	Mucoromycota	KP311420	641/641 (100)	0.99	1.10	0.31	0.98	0.78	1.53	0.97	1.02	0.30	1.97	0.80	1.26	1.02
*Saitozyma podzolica*	Basidiomycota	KY320605	511/511 (100)	1.00	1.56	0.11	1.48	0.34	1.75	0.26	1.97	0.21	1.14	0.41	1.65	0.99
Unidentified sp. 3993_29	Ascomycota	FN393100	546/550 (99)	0.34	0.12	0.34	0.56	1.33	0.40	1.90	1.01	0.91	0.41	1.22	0.57	0.92
*Pseudogymnoascus* sp. 3993_33	Ascomycota	KY977601	562/562 (100)	0.19	3.84	3.21	1.01	0.33	1.39	0.22	0.59	0.46	0.67	0.48	1.33	0.87
*Penicillium* sp. 3993_47	Ascomycota	MK226541	579/579 (100)	0.29	2.54	0.04	1.05	0.29	1.04	0.43	2.24	0.12	0.80	0.30	1.54	0.87
*Mortierella* sp. 3993_38	Mucoromycota	HG935763	640/640 (100)	1.43	1.77	-	0.74	0.34	1.42	0.39	1.36	0.15	1.29	0.52	1.28	0.87
*Trichoderma crassum*	Ascomycota	NR134370	610/610 (100)	0.97	0.98	0.15	0.74	0.51	0.88	0.79	1.22	1.64	0.70	0.76	0.93	0.84
*Fusarium solani*	Ascomycota	MF782768	562/562 (100)	0.14	3.87	0.08	1.09	0.31	1.65	0.31	0.82	0.12	1.45	0.25	1.57	0.86
*Pestalotiopsis* sp. 3993_40	Ascomycota	KT963804	588/588 (100)	1.65	0.70	0.08	0.52	0.26	0.92	0.26	1.77	0.49	1.68	0.53	1.12	0.80
*Fusarium* sp. 3993_41	Ascomycota	MH550484	559/559 (100)	1.03	0.70	1.11	1.24	0.41	1.19	0.29	1.18	0.18	0.44	0.51	1.04	0.76
*Sebacina* sp. 3993_35	Basidiomycota	JX844771	641/643 (99)	0.03	0.00	0.27	0.03	3.67	0.00	0.06	0.48	0.06	-	1.28	0.14	0.75
Total of 30 OTUs				46.51	39.84	52.33	43.31	73.83	39.56	65.78	41.48	44.15	22.50	62.03	38.95	51.3

^a^ Sequence similarity column shows base pairs compared between the query sequence and the reference sequence in the NCBI databases, with the percentage of the sequence similarity in parentheses.

**Table 4:** Occurrence and relative abundance of the 30 most common oomycete OTUs (shown as a proportion of all of the high-quality fungal sequences) in the roots and soil of the five tree species that were bare-root cultivated in the forest nurseries. The data from the different forest nurseries are combined.

OTU	Reference	Similarity % ^a^	*Alnus glutinosa*		*Betula pendula*		*Picea abies*		*Pinus sylvestris*		*Quercus robur*		All	All	Total %
			Roots %	Soil %	Roots %	Soil %	Roots %	Soil %	Roots %	Soil %	Roots %	Soil %	Roots %	Soil %	
*Pythium ultimum* var. *ultimum*	AY598657	917/917 (100)	0.258	0.559	0.076	1.435	0.048	0.829	0.067	1.349	0.030	1.554	0.094	1.161	0.59
*Pythium heterothallicum*	AY598654	882/889 (99)	0.143	0.210	0.076	0.545	-	0.251	0.008	0.812	0.030	0.829	0.038	0.534	0.27
*Pythium spiculum*	HQ643790	972/978 (99)	0.072	0.047	0.038	0.040	0.032	0.088	1.243	0.114	-	-	0.423	0.068	0.26
*Pythium sylvaticum*	AY598645	997/999 (99)	0.172	0.326	0.496	0.385	0.088	0.138	0.243	0.057	0.030	0.466	0.177	0.238	0.21
*Pythium irregulare*	AY598702	1026/1029 (99)	0.043	0.280	0.076	0.133	0.128	0.301	0.151	0.114	0.091	0.207	0.113	0.198	0.15
*Peronospora* sp. 3993_148	MF372507	803/852 (94)	-	-	-	-	-	-	-	-	2.765	-	0.244	-	0.13
*Pythium intermedium*	KU211482	957/959 (99)	-	0.489	0.038	0.133	-	0.038	0.059	0.057	-	0.026	0.021	0.124	0.07
*Pythium amasculinum*	AY598671	856/857 (99)	0.029	-	-	0.159	0.008	0.025	0.008	0.023	-	0.363	0.011	0.093	0.05
*Pythium* sp. 3993_349	KU211471	894/907 (99)	0.014	0.047	-	0.053	0.024	-	0.008	0.057	-	0.363	0.013	0.077	0.04
Unidentified sp. 3993_508	MF570293	101/119 (85)	0.029	0.047	0.038	0.040	0.008	0.050	0.025	0.069	0.030	0.104	0.021	0.059	0.04
*Pythium acanthicum*	AY598617	858/859 (99)	0.014	-	-	0.013	0.040	0.050	0.017	0.057	0.030	0.104	0.024	0.043	0.03
*Pythium apiculatum*	HQ643443	948/954 (99)	0.072	0.023	-	0.120	-	0.063	-	0.011	0.061	-	0.019	0.049	0.03
*Pythium rostratifingens*	KU211363	1053/1064 (99)	0.115	0.023	0.038	0.066	-	-	-	0.034	0.030	0.104	0.027	0.040	0.03
*Phytopythium citrinum*	HM061322	852/857 (99)	-	-	0.496	-	-	-	0.008	-	0.030	-	0.040	-	0.02
Unidentified sp. 3993_709	KJ716873	724/865 (84)	-	-	-	-	0.016	-	0.109	0.011	-	-	0.040	0.003	0.02
*Pythium pleroticum*	AY598642	958/959 (99)	-	0.093	-	-	-	0.063	-	-	-	0.078	-	0.037	0.02
*Pythium* sp. 3993_1159	AY598639	940/966 (97)	0.086	-	-	-	-	0.075	-	-	-	-	0.016	0.019	0.02
Unidentified sp. 3993_729	HQ643756	226/240 (94)	-	-	-	-	0.064	0.013	0.008	0.023	-	-	0.024	0.009	0.02
*Pythium rostratifingens*	KU209835	962/969 (99)	-	0.047	-	0.053	-	0.050	-	-	-	-	-	0.031	0.01
*Pythium* sp. 3993_1163	AY598696	993/1036 (96)	0.043	-	-	-	-	0.013	-	0.034	-	0.078	0.008	0.022	0.01
Unidentified sp. 3993_943	MH671329	870/883 (99)	-	-	-	-	0.048	0.025	-	0.011	-	0.026	0.016	0.012	0.01
Unidentified sp. 3993_1191	KF318041	606/754 (80)	-	0.023	-	-	-	-	-	0.046	-	0.104	-	0.028	0.01
*Phytophthora fragariae*	KJ755093	896/905 (99)	0.029	0.116	0.038	-	-	-	-	-	-	-	0.008	0.015	0.01
*Phytophthora pseudosyringae*	EU074793	838/848 (99)	-	-	-	-	-	-	-	0.011	-	0.181	-	0.025	0.01
*Pythium violae*	AY598717	1001/1006 (99)	0.043	0.023	-	0.013	-	0.025	-	0.011	-	-	0.008	0.015	0.01
Unidentified sp. 3993_1954	LC176476	143/157 (91)	0.057	-	-	0.053	-	-	-	-	-	-	0.011	0.012	0.01
Unidentified sp. 3993_981	KF318041	602/754 (80)	-	0.023	-	0.040	-	-	-	-	-	0.104	-	0.025	0.01
*Pythium* sp. 3993_1117	JF431913	804/842 (95)	-	-	-	0.013	-	0.050	-	-	-	0.052	-	0.022	0.01
Unidentified sp. 3993_1171	KJ716873	797/854 (93)	0.014	-	-	0.040	-	-	-	-	-	0.078	0.003	0.019	0.01
*Hyaloperonospora brassicae*	MG757782	940/943 (99)	-	-	-	-	-	-	0.050	-	-	-	0.016	-	0.01
Total of 30 OTUs			1.232	2.377	1.412	3.336	0.503	2.148	2.007	2.905	3.130	4.816	1.414	2.977	2.14

^a^ Sequence similarity column shows base pairs compared between the query sequence and the reference sequence in the NCBI databases, with the percentage of the sequence similarity in parentheses.

## Data Availability

The data presented in this study are available in Supplementary Material, Table S1.

## Supplementary Materials

The following are available online at https://www.mdpi.com/2076-2607/9/1/150/s1. Table S1: Relative abundance of fungal and oomycete OTUs sequenced from the root and soil samples of the five tree species bare-root cultivated in forest nurseries in Lithuania.
